# Supplementary material for: Unsupervised cross domain adaptive anomaly detection network for Internet of Things traffic
Source: PLoS One. 2026 Apr 2;21(4):e0344009. doi: 10.1371/journal.pone.0344009 (PMC13046153; doi:10.1371/journal.pone.0344009)
Supplement: Attachment — Submitted filename: replyletter.pdf [file pone.0344009.s001.pdf]

Replies to Reviews of “Unsupervised Cross Domain Adaptive Anomaly Detection Network for Internet of Things Traffic” (Paper ID PONE-D-25-28923 of *PLOS One*)

Tiange Yuan, Di Zhai, Anchao Li

11/17/2025

Abstract

We would like to express our appreciation to the editors and reviewers for generously dedicating their time and expertise to process our manuscript. We also want to acknowledge the invaluable feedback provided by the reviewers, which has significantly contributed to improving the clarity and quality of our work. We will carefully address their comments in this letter and the revised manuscript, ensuring that we have addressed every concern.

In this letter, the text in *italic* represents feedback from the AE or reviewers. Following each comment, our answers are provided under the “**Response**” header. Modifications made in the manuscript are highlighted in GREEN. We trust this letter adequately addresses the concerns of the AE and reviewers.

1 Replies to Reviewer 1

1. Terminology consistency (“unsupervised” + few-shot tuning)

Explicitly state that a small set of normal target samples is used for fine-tuning; align wording across Abstract, Introduction, and Method.

Response: We appreciate the reviewer’s helpful suggestion regarding the consistency between the terms “unsupervised” and the usage of few-shot adaptation samples. The CDA-ADN framework is still considered *unsupervised* because no labeled anomaly samples from the target domain are required at any stage of training. During the adaptation step, we only utilize a very small number of normal target-domain samples, which is a standard and widely adopted assumption in unsupervised domain adaptation and one-class anomaly detection. These normal samples do not introduce supervised anomaly labels, and thus do not contradict the unsupervised nature of the model.

To ensure clarity and consistency, we have revised the Abstract, Introduction, and Method sections to explicitly emphasize:

1. CDA-ADN does not require any labeled anomaly samples from the target domain.
2. Only a very small number of normal target-domain samples are used for lightweight fine-tuning during adaptation.
3. The entire framework remains unsupervised with respect to anomaly detection.

These revisions ensure consistent terminology throughout the manuscript and remove any potential ambiguity regarding the training setup.

The following are the revisions made in the **Abstract** to clarify the unsupervised setting and the use of a few normal target-domain samples:

In the first stage the model learns general normal behavior on a source environment using only normal samples. In the second stage a very small number of unlabeled normal samples from the target environment are used for lightweight fine tuning of the adaptation layers, without requiring any labeled anomaly samples in the target domain.

The following is the revision made in the **Introduction** to clarify the unsupervised cross-domain setting:

In this work, we adopt an unsupervised cross-domain anomaly detection setting where no labeled anomaly samples from the target environment are required. CDA-ADN learns general normal behavioral patterns from a source domain and subsequently adapts to a new target domain using only a very small number of unlabeled normal samples. These normal samples are employed solely to calibrate domain-specific components and do not introduce supervised anomaly information. This setting aligns with widely accepted assumptions in unsupervised domain adaptation and one-class anomaly detection, ensuring that the proposed framework remains fully unsupervised with respect to anomaly identification.

The following is the revision made in the **Introduction** to refine the description of the contribution involving few-shot target samples:

We further clarify our contribution by emphasizing that the proposed two-stage optimization strategy includes a lightweight fine-tuning step using only a minimal set of normal target-domain samples. This fine-tuning does not rely on any labeled anomalies and is performed exclusively to adapt the latent-guided affine transformation layers to the target distribution. As such, the method preserves the unsupervised nature of the framework while enabling effective cross-domain alignment in label-scarce Internet of Things environments.

The following is the revision made in the **Method** section to unify the terminology regarding unsupervised training and few-shot adaptation:

At the beginning of the training pipeline, CDA-ADN performs source-domain learning using only normal traffic sequences, without requiring any labels from the target domain. During the subsequent domain adaptation stage, a very small number of unlabeled normal target-domain samples are used exclusively to calibrate the domain-specific transformation layers. No labeled anomaly data from the target domain are employed at any point. This refinement ensures terminological consistency and clearly distinguishes our unsupervised setting from semi-supervised alternatives.

The following is the revision made in the **Method** section to clarify the role of few-shot normal samples in the problem formulation:

In the problem formulation, we explicitly state that the few-shot fine-tuning mechanism operates only on a limited subset of unlabeled normal samples from the target domain. This process adjusts the latent-guided affine adaptation layers to account for domain-specific characteristics while preserving the unsupervised nature of anomaly detection, as no anomaly labels from the target domain are used.

2. Loss details in one place Briefly specify how the domain-adaptation/Wasserstein term is instantiated and list key contrastive-loss hyperparameters (e.g., margin m, balance alpha).

Response: We sincerely thank the reviewer for the helpful suggestion to consolidate and clarify the loss definitions. In the revised manuscript, we have added a dedicated subsection in the Method section that summarizes all components of the training objective in one place. The revised text now (1) specifies how the Wasserstein domain-adaptation term is instantiated, including the critic network and gradient penalty formulation; (2) explicitly lists the key hyperparameters used in the local and global contrastive losses, such as the margin m, balance coefficient α, and temperature τ; and (3) presents the complete total loss function used during training. These additions improve clarity and reproducibility of the proposed framework.

The following is the revision made in the **Method** section to consolidate the loss definitions and provide explicit formulations and hyperparameters:

Overall Training Objective

To ensure clarity and reproducibility, we summarize all loss components of CDA-ADN in a unified formulation. The domain-adaptation loss is implemented using the Wasserstein-1 distance with a gradient-penalized critic network. Given source-domain latent representations z_s and target-domain latent representations z_t, the critic $D(\cdot)$ is trained to maximize

$$\mathcal{L}_{\text{W}} = \mathbb{E}_{z_s}[D(z_s)] - \mathbb{E}_{z_t}[D(z_t)],$$

while enforcing 1-Lipschitz continuity via the gradient penalty

$$\mathcal{L}_{\text{GP}} = \lambda \, \mathbb{E}_{\hat{z}} \left(\|\nabla_{\hat{z}} D(\hat{z})\|_2 - 1 \right)^2,$$

where $\hat{z}$ is sampled along straight lines between z_s and z_t. The domain-adaptation objective is therefore

$$\mathcal{L}_{\text{DA}} = \mathcal{L}_{\text{W}} + \mathcal{L}_{\text{GP}}.$$

For contrastive learning, we employ margin-based losses at both the local time-step level and the global sequence level. For a pair of latent features (h_i, h_j) with pseudo-label $y_{ij} \in \{0, 1\}$ indicating positive or negative relation, the contrastive loss is

$$\mathcal{L}_{\text{con}}(h_i, h_j) = y_{ij}\|h_i - h_j\|_2^2 + (1 - y_{ij})\max(0, m - \|h_i - h_j\|_2)^2,$$

where m is the contrastive margin. Local and global contrastive objectives are combined as

$$\mathcal{L}_{\text{CL}} = \alpha\mathcal{L}_{\text{local}} + (1 - \alpha)\mathcal{L}_{\text{global}},$$

with balance coefficient α and temperature parameter τ used in the similarity normalization process.

Finally, the total training objective integrates the variational reconstruction loss $\mathcal{L}_{\text{VAE}}$, the domain-adaptation loss $\mathcal{L}_{\text{DA}}$, and the multi-granularity contrastive loss $\mathcal{L}_{\text{CL}}$:

$$\mathcal{L}_{\text{total}} = \mathcal{L}_{\text{VAE}} + \beta\mathcal{L}_{\text{DA}} + \gamma\mathcal{L}_{\text{CL}},$$

where β and γ are weighting coefficients. Table **??** provides the key hyperparameters used in the contrastive and domain-adaptation losses for completeness.

3. Training recipe summary box Add seeds and number of trials to the already-reported learning rate, batch size, and epochs; present them in a compact "Training Setup" box or table for reproducibility.

Response: We sincerely thank the reviewer for the constructive suggestion regarding the consolidation of the training configuration. In the revised manuscript, we have added a compact "Training Setup" summary box that includes all relevant hyperparameters, including random seeds and the number of repeated trials. This enhances the clarity and reproducibility of the experimental protocol and ensures that all training-related settings are presented in a single, easily accessible location.

The following is the revision made in the **Experimental Setup** section to incorporate a unified training configuration summary:

Table 1: Training Setup Summary

**Parameter**	**Value**
Optimizer	Adam
Learning rate	1×10^{-3}
Batch size	128
Epochs	100
Random seeds	2025
Number of trials	5
Hardware	NVIDIA A100

4. Metrics: add two standard curves Keep Accuracy/MCC/Sensitivity, but add AUC-ROC and AUC-PR for each transfer setting to provide threshold-independent views.

Response: We sincerely thank the reviewer for the constructive suggestion on evaluation metrics. In the revised manuscript, we have retained Accuracy, MCC, and Sensitivity, and additionally report AUC-ROC and AUC-PR for all compared methods on the target domain. Specifically, Table 5 has been updated to include two new columns for AUC-ROC and AUC-PR with mean and standard deviation, and a short paragraph has been added to discuss these threshold-independent metrics. These changes provide a more comprehensive and threshold-agnostic assessment of anomaly detection performance.

The following are the revisions made in the **Experimental Results and Analysis** section to incorporate AUC-ROC and AUC-PR:

The performance of CDA-ADN and baseline methods on the target domain is summarized in Table 5. To provide a more comprehensive evaluation, we report the mean and standard deviation across multiple runs for five metrics: Accuracy, MCC, Sensitivity, AUC-ROC, and AUC-PR. While Accuracy, MCC, and Sensitivity reflect thresholded classification performance, AUC-ROC and AUC-PR offer threshold-independent views that are particularly informative under class imbalance.

Table 2: Performance comparison on the target domain. Values are reported as mean ± standard deviation over five independent runs.

Model	Accuracy (%)	MCC	Sensitivity	AUC-ROC	AUC-PR
AE	76.5 ± 1.2	0.52 ± 0.03	0.57 ± 0.05	0.75 ± 0.03	0.28 ± 0.05
VAE	81.3 ± 1.5	0.62 ± 0.04	0.66 ± 0.06	0.87 ± 0.02	0.51 ± 0.04
CDA-ADN	92.8 ± 0.7	0.80 ± 0.02	0.88 ± 0.03	0.92 ± 0.01	0.64 ± 0.03

The threshold-independent metrics AUC-ROC and AUC-PR are consistent with the above trends. CDA-ADN obtains the highest AUC-ROC (0.92 ± 0.01) and AUC-PR (0.64 ± 0.03), clearly surpassing VAE (0.87 ± 0.02 and 0.51 ± 0.04) and AE (0.75 ± 0.03 and 0.28 ± 0.05). These improvements confirm that CDA-ADN not only performs better at a fixed decision threshold, but also dominates the baselines across a broad range of thresholds, which is crucial under highly imbalanced anomaly detection conditions.

5. Thresholding and decision rule Add one sentence clarifying how detection thresholds are chosen (for example, selected on a validation split using source/normal data).

Response: The authors sincerely thank the reviewer for pointing out the need to clarify the decision rule. In the revised manuscript, the thresholding strategy for turning continuous anomaly scores into binary decisions has been explicitly specified in the Experimental Setup section. In particular, all methods use a common reconstruction based anomaly score, and a single decision threshold is selected on a held out validation subset that contains only normal samples from the source domain by taking the 95th percentile of validation scores. This threshold is then kept fixed for all transfer evaluations on the target domain, without any tuning on target data. This clarification makes the reported Accuracy, MCC and Sensitivity metrics fully reproducible and consistent with the unsupervised cross domain setting.

The following sentence has been added in the **Experimental Setup** subsection to describe the thresholding and decision rule:

Anomalies are decided by thresholding a reconstruction based anomaly score. The decision threshold τ is selected on a held out validation subset that contains only normal samples from the source domain by setting τ to the 95th percentile of the validation scores.

6. Table 1 caption polish State the number of runs and include 95% confidence intervals (or mean ± std along with the sample count n) to make improvements easy to interpret.

Response: We sincerely thank the reviewer for the helpful suggestion on improving the interpretability of Table 5. In the revised manuscript, we have explicitly revised the caption of Table 5 to state that all reported values are mean ± standard deviation over five independent runs. In addition, the Experimental Setup subsection now summarizes this setting in the Training Setup Summary table through the entry "Number of trials = 5", and the Experimental Results and Analysis section explicitly states that all metrics are averaged over these five random seeds.

The following are the revisions made in the caption of Table 5 to clarify the number of runs used for mean ± std:

Table 3: Performance comparison on the target domain. Values are reported as mean ± standard deviation over five independent runs.

Model	Accuracy (%)	MCC	Sensitivity	AUC-ROC	AUC-PR
AE	76.5 ± 1.2	0.52 ± 0.03	0.57 ± 0.05	0.75 ± 0.03	0.28 ± 0.05
VAE	81.3 ± 1.5	0.62 ± 0.04	0.66 ± 0.06	0.87 ± 0.02	0.51 ± 0.04
CDA-ADN	92.8 ± 0.7	0.80 ± 0.02	0.88 ± 0.03	0.92 ± 0.01	0.64 ± 0.03

7. Tiny sensitivity check in ablation. Include a brief stability check varying the local/global balance alpha or the contrastive margin; a short text/table in the appendix is sufficient.

Response: We thank the reviewer for the constructive suggestion regarding the stability of the proposed method with respect to the contrastive learning hyperparameters. In the revised manuscript, we have added a new subsection in the Experiments section that reports a sensitivity analysis of the balance coefficient α between local and global contrastive terms and the margin m in the contrastive loss. We vary α and m over a relatively wide range and summarize the corresponding accuracy on the target domain in a compact table. The results show that CDA-ADN maintains high and stable accuracy for moderate values of α and m, and only exhibits noticeable degradation when these hyperparameters take extreme values. This analysis supports the robustness of the proposed framework to the choice of contrastive hyperparameters.

The following are the revisions made in the **Experiments** section, where a new subsection entitled "**Sensitivity Analysis of Hyperparameters**" has been added to report the sensitivity analysis of the contrastive hyperparameters:

To further assess the robustness of CDA-ADN with respect to the contrastive learning objective, we perform a sensitivity analysis on two key hyperparameters: the balance coefficient α between local and global contrastive terms, and the margin m that controls the separation between positive and negative pairs in the contrastive loss. We vary α and m over a relatively wide range while keeping all other settings fixed, and report the classification accuracy on the target domain. The default configuration used in our main experiments is $\alpha = 0.5$ and $m = 0.5$.

Table 4: Sensitivity of CDA-ADN to the balance coefficient α and margin m on the target domain (accuracy, mean ± std over five runs).

α	0.2	0.5	0.5	0.8	0.8	0.1	0.9
m	0.3	0.3	0.5	0.5	0.8	0.1	1.0
Accuracy (%)	92.1 ± 0.8	92.6 ± 0.7	92.8 ± 0.7	92.4 ± 0.8	92.0 ± 0.9	90.9 ± 1.1	90.7 ± 1.2

As shown in Table 4, CDA-ADN maintains consistently high accuracy in a broad and practically reasonable range of α and m. For $\alpha \in [0.2, 0.8]$ and $m \in [0.3, 0.8]$, the accuracy remains around 92%–93%, close to the default setting (92.8%), with only minor fluctuations. When the hyperparameters take more extreme values, such as $(\alpha, m) = (0.1, 0.1)$ or $(0.9, 1.0)$, the accuracy decreases more noticeably (to about 90.7%–90.9%), indicating that excessive emphasis on either local or global contrastive structure, or an overly small/large margin, can harm performance. Overall, these results demonstrate that CDA-ADN is robust to the choice of contrastive hyperparameters within a wide range around the default configuration.

8. Figure/notation alignment Ensure symbols in the architecture figure match the equations and add dimensionalities in the caption (latent size, sequence length).

Response: We thank the reviewer for pointing out the inconsistency in the notation of the latent representations between the equations and the architectural description. In the revised manuscript, the latent representation is now denoted consistently by **c** throughout the Method section, including the problem formulation, the contrastive loss definition, and the Wasserstein-based domain adaptation loss.

The following are the revisions made in the **Method** section to unify the notation of the latent representations:

The objective is to learn a function $F_\theta : \mathbb{R}^{T \times d} \to \mathcal{H}$ that transforms sequences into a latent space $\mathbf{c} \in \mathcal{H}$, where normal and anomalous behaviors are well-separated.

The following are the revisions made in the **Method** section to unify the notation of the latent representations:

Anomalies are identified through reconstruction errors and deviations within the latent space. Given an encoded representation **c**, the model reconstructs the input sequence $\hat{\mathbf{X}}$, and the reconstruction loss is computed as

$$\mathcal{L}_{rec} = \frac{1}{T} \sum_{t=1}^{T} \|\mathbf{x}_t - \hat{\mathbf{x}}_t\|^2.$$

The following are the revisions made in the **Method** section to unify the notation of the latent representations:

To further improve feature discrimination, multi-granularity contrastive learning is introduced, where representations of similar samples are pulled together while dissimilar ones are pushed apart:

$$\mathcal{L}_{ctr} = \sum_{(i,j)\in\mathcal{P}} \log\sigma\big(-d(\mathbf{c}_i,\mathbf{c}_j)\big) + \sum_{(i,j)\in\mathcal{N}} \log\sigma\big(d(\mathbf{c}_i,\mathbf{c}_j)-m\big),$$

where $\mathcal{P}$ and $\mathcal{N}$ denote the sets of positive (similar) and negative (dissimilar) pairs, $d(\mathbf{c}_i,\mathbf{c}_j)$ represents the distance between representations, and m is a margin parameter.

The following are the revisions made in the **Method** section to unify the notation of the latent representations:

To enforce cross-domain feature alignment, a Wasserstein distance-based domain adaptation loss is used:

$$\mathcal{L}_{wd} = \inf_{\gamma\in\Gamma(P_s,P_t)} \mathbb{E}_{(\mathbf{c}_s,\mathbf{c}_t)\sim\gamma}\big[\,\|\mathbf{c}_s-\mathbf{c}_t\|\,\big],$$

where $\Gamma(P_s,P_t)$ denotes the set of all joint distributions with marginals P_s and P_t. This loss minimizes distributional discrepancies between $\mathcal{D}_s$ and $\mathcal{D}_t$, enabling better generalization in the target domain.

9. Data and code pointers Near the Data Availability statement, add exact preprocessing details (window length, normalization) and, if possible, a link to configs/code.

Response: We thank the reviewer for the helpful suggestion regarding data and code accessibility. In the revised manuscript, detailed descriptions of the preprocessing pipeline (including sequence windowing and feature normalization) have been added to the Experimental Setup subsection, and the corresponding implementation and configuration files will be released on GitHub.

The following are the revisions made in the **Experiments** section to clarify the preprocessing pipeline and input normalization:

For both datasets, continuous traffic features are standardized using z-score normalization with mean and standard deviation computed on the source-domain training split and then reused for all target-domain samples. The normalized streams are segmented into fixed-length windows of $T = 128$ time steps with a stride of $S = 64$, which serve as the multivariate inputs to CDA-ADN.

10. Below articles can be cited:

Response: We thank the reviewer for recommending these relevant and recent references. In the revised manuscript, we will incorporate these citations at appropriate places in the text to better reflect the latest advances related to our work and strengthen the discussion in the relevant sections.

2 Replies to Reviewer 2

The proposal of combining conditional variational sequence encoding with multi-granularity contrastive learning for unsupervised cross-domain IoT anomaly detection addresses a clear gap in handling domain shifts without requiring labeled target data. However, the paper could strengthen its novelty claim by thoroughly contrasting CDA-ADN with the latest Transformer-based or graph-based anomaly detectors noted in related work. Clarifying how CDA-ADN advances beyond or complements these emerging architectures would enhance the technical contribution.

Response: We thank the reviewer for pointing out the importance of a clearer comparison with recent Transformer-based and graph-based anomaly detectors. In the revised manuscript, the experimental comparison has been extended to include representative Transformer-based and graph-based baselines for IoT traffic anomaly detection in the Experiments section, and the corresponding discussion highlights how CDA-ADN complements these architectures through its conditional variational sequence encoding and multi-granularity contrastive learning under fully unsupervised cross-domain settings.

The following are the revisions made in the **Baseline Methods** subsection to incorporate AT and GDN:

- **Autoencoder (AE) [1]**: An unsupervised deep learning model that reconstructs input data and detects anomalies based on reconstruction errors.
- **Variational Autoencoder (VAE) [2]**: A probabilistic extension of AE that learns a structured latent space, improving anomaly detection generalization.
- **Anomaly Transformer (AT) [3]**: A Transformer-based time-series anomaly detector that models long-range temporal dependencies and measures association discrepancy between time steps to highlight abnormal patterns.
- **Graph Deviation Network (GDN) [4]**: A graph neural network based model that leverages an adaptive sensor graph to capture feature correlations and detects anomalies by measuring deviations from learned graph-structured normal patterns.

The following are the revisions made in the **Experimental Results and Analysis** subsection to incorporate AT and GDN:

Table 5: Performance comparison on the target domain. Values are reported as mean ± standard deviation over five independent runs.

Model	Accuracy (%)	MCC	Sensitivity	AUC-ROC	AUC-PR
AE	76.5 ± 1.2	0.52 ± 0.03	0.57 ± 0.05	0.75 ± 0.03	0.28 ± 0.05
VAE	81.3 ± 1.5	0.62 ± 0.04	0.66 ± 0.06	0.87 ± 0.02	0.51 ± 0.04
AT	87.5 ± 1.0	0.70 ± 0.03	0.79 ± 0.04	0.90 ± 0.02	0.58 ± 0.04
GDN	88.6 ± 0.9	0.73 ± 0.03	0.81 ± 0.04	0.91 ± 0.01	0.60 ± 0.03
CDA-ADN	92.8 ± 0.7	0.80 ± 0.02	0.88 ± 0.03	0.92 ± 0.01	0.64 ± 0.03

Table 5 shows that CDA-ADN consistently outperforms all baselines, including reconstruction-based models (AE, VAE), Transformer-based AT, and graph-based GDN. In terms of Accuracy, CDA-ADN achieves the best result (92.8 ± 0.7%), surpassing the strongest baseline GDN (88.6 ± 0.9%) and AT (87.5 ± 1.0%). This demonstrates that CDA-ADN maintains superior detection performance under cross-domain distribution shifts.

MCC further confirms the advantage of CDA-ADN on imbalanced anomaly detection. CDA-ADN obtains the highest MCC (0.80 ± 0.02), improving over GDN (0.73 ± 0.03) and AT (0.70 ± 0.03), and substantially outperforming VAE (0.62 ± 0.04) and AE (0.52 ± 0.03). This indicates that CDA-ADN better balances false alarms and missed detections, which is critical in real-world IoT security settings.

In terms of Sensitivity, CDA-ADN also ranks first (0.88 ± 0.03), exceeding GDN (0.81 ± 0.04) and AT (0.79 ± 0.04). This gap suggests that CDA-ADN is more effective at capturing abnormal patterns in the target domain. Combined with the improvements in AUC-ROC and AUC-PR reported above, these results confirm that CDA-ADN not only performs better at a fixed threshold, but also provides consistently stronger discrimination across a wide range of decision thresholds under severe class imbalance.

The description of the conditional variational encoder architecture is somewhat high-level. Including more details such as the exact GRU configuration, latent space dimensionality, and the weighting scheme for the KL divergence term would improve reproducibility.

Response: We appreciate the reviewer's suggestion to provide a more detailed and reproducible description of the conditional variational sequence encoder. In the revised manuscript, additional implementation details have been added in the Method section to clarify the GRU configuration, the dimensionality of the latent space, and the weighting scheme used for the KL divergence term.

The following are the revisions made in the **Conditional Variational Sequence Encoding** subsection to specify the architecture and training schedule of the conditional variational sequence encoder.

Concretely, the encoder uses a single layer GRU with hidden size $H = 128$ that processes the input sequence $\mathbf{X}$ together with the contextual vector $\mathbf{c}_{\text{ctx}}$ and produces a final hidden state $\mathbf{h}_T \in \mathbb{R}^H$. Two linear projection heads f_ψ and g_ψ map $\mathbf{h}_T$ to the mean and variance of a $d_c = 64$ dimensional latent variable $\mathbf{c}$, which defines the conditional posterior $q_\psi(\mathbf{c} \mid \mathbf{X}, \mathbf{c}_{\text{ctx}})$ used in the reparameterization step. The KL divergence term is weighted by a coefficient λ_{kl} that is linearly increased from 0 to 10^{-3} during the first 20 training epochs

and then kept fixed, which stabilizes early optimization while still enforcing a well structured latent space at convergence.

Similarly, the dynamics of the input-output adaptation layers (latent-guided affine transformations) would benefit from equations or pseudocode to clearly describe how domain alignment is performed.

Response: We thank the reviewer for pointing out the need for a clearer description of the latent-guided input-output adaptation mechanism. In the revised manuscript, we add explicit equations in the **Dynamic Domain Adaptation Layers** subsection to clarify how the affine transformations are conditioned on the latent representations and how they perform cross-domain alignment.

The following are the revisions made in the **Dynamic Domain Adaptation Layers** subsection to explicitly formalize how the affine transformations are parameterized by the latent representations and how domain alignment is performed.

Concretely, given the time-dependent latent variables $\{\mathbf{c}_t\}_{t=1}^{T}$, we first aggregate them into a sequence-level descriptor

$$\bar{\mathbf{c}} = \frac{1}{T}\sum_{t=1}^{T}\mathbf{c}_t.$$

For each domain $d \in \{s, t\}$, the input adaptation layer predicts feature-wise scaling and shifting vectors from $\bar{\mathbf{c}}$

$$\boldsymbol{\gamma}_d(\bar{\mathbf{c}}) = \mathbf{W}_d^{\gamma}\bar{\mathbf{c}} + \mathbf{b}_d^{\gamma}, \qquad \boldsymbol{\beta}_d(\bar{\mathbf{c}}) = \mathbf{W}_d^{\beta}\bar{\mathbf{c}} + \mathbf{b}_d^{\beta},$$

and the adapted input sequence for domain d is computed as

$$\tilde{\mathbf{x}}_t^{(d)} = \boldsymbol{\gamma}_d(\bar{\mathbf{c}}) \odot \mathbf{x}_t^{(d)} + \boldsymbol{\beta}_d(\bar{\mathbf{c}}), \quad t = 1, \ldots, T.$$

Similarly, the output adaptation layer uses $\bar{\mathbf{c}}$ to parameterize domain-specific normalization statistics

$$\boldsymbol{\mu}_d(\bar{\mathbf{c}}) = \mathbf{W}_d^{\mu}\bar{\mathbf{c}} + \mathbf{b}_d^{\mu}, \qquad \boldsymbol{\sigma}_d(\bar{\mathbf{c}}) = \mathrm{SoftPlus}\big(\mathbf{W}_d^{\sigma}\bar{\mathbf{c}} + \mathbf{b}_d^{\sigma}\big),$$

and aligns reconstructed features across domains via

$$\hat{\mathbf{x}}_t^{(d)} = \frac{\hat{\mathbf{x}}_t - \boldsymbol{\mu}_d(\bar{\mathbf{c}})}{\boldsymbol{\sigma}_d(\bar{\mathbf{c}})}, \quad d \in \{s, t\}.$$

These latent-guided affine transformations explicitly couple the shared latent representation with domain-wise feature normalization, making the dynamics of the input-output adaptation layers and the resulting cross-domain alignment more transparent and reproducible.

While the multi-granularity contrastive learning approach is promising, the manuscript should elaborate on the specific loss functions used at local and global levels and how these losses are combined during optimization. This would ensure clarity on how the model balances separating normal from anomalous patterns while preserving domain-invariant features.

Response: We appreciate the reviewer's suggestion to further clarify the instantiation of the local and global contrastive losses. In the revised manuscript, we explicitly define the loss functions at both granularities and describe how they are combined into a single multi-granularity contrastive objective, which is then weighted in the overall training loss to balance anomaly separation and domain-invariant representation learning.

The following are the revisions made in the **Multi-Granularity Contrastive Learning and Domain Adaptation** subsection to provide a precise formulation of the local and global contrastive losses and their role in the optimization objective.

Concretely, let $d(\cdot, \cdot)$ denote the Euclidean distance in the latent space. For the local level, we compute distances between time-step representations and define the contrastive loss as

$$\mathcal{L}_{\text{local}} = -\frac{1}{|\mathcal{P}_{\text{local}}|}\sum_{(i,j)\in\mathcal{P}_{\text{local}}}\log\sigma\big(-d_{ij}^{\text{loc}}\big) - \frac{1}{|\mathcal{N}_{\text{local}}|}\sum_{(i,j)\in\mathcal{N}_{\text{local}}}\log\sigma\big(d_{ij}^{\text{loc}} - m\big),$$

where $d_{ij}^{\text{loc}} = d(\mathbf{c}_i^{\text{loc}}, \mathbf{c}_j^{\text{loc}})$ denotes the distance between two time-step embeddings, $\sigma(\cdot)$ is the sigmoid function, and m is a margin that encourages negatives to be separated beyond a minimum distance. At the global level, we construct sequence-wise positive and negative pairs and use the same logistic form,

$$\mathcal{L}_{\text{global}} = -\frac{1}{|\mathcal{P}_{\text{global}}|} \sum_{(i,j)\in\mathcal{P}_{\text{global}}} \log \sigma\big(-d_{ij}^{\text{glob}}\big) - \frac{1}{|\mathcal{N}_{\text{global}}|} \sum_{(i,j)\in\mathcal{N}_{\text{global}}} \log \sigma\big(d_{ij}^{\text{glob}} - m\big),$$

where $d_{ij}^{\text{glob}} = d(\mathbf{c}_i^{\text{glob}}, \mathbf{c}_j^{\text{glob}})$ measures distances between sequence-level latent representations. The overall multi-granularity contrastive objective is given by

$$\mathcal{L}_{ctr} = \alpha\mathcal{L}_{\text{local}} + (1-\alpha)\mathcal{L}_{\text{global}},$$

with $\alpha \in [0, 1]$ controlling the trade-off between local temporal consistency and global sequence-level discrimination. During training, $\mathcal{L}_{ctr}$ is incorporated into the total loss as

$$\mathcal{L} = \lambda_{rec}\mathcal{L}_{rec} + \lambda_{kl}\mathcal{L}_{kl} + \lambda_{ctr}\mathcal{L}_{ctr} + \lambda_{wd}\mathcal{L}_{wd},$$

where λ_{ctr} determines the relative strength of the contrastive objective. This explicit formulation clarifies how the model jointly separates normal and anomalous patterns at different granularities while preserving domain-invariant features through the Wasserstein distance term and the shared latent space.

The justification for using Wasserstein distance in domain adaptation is briefly mentioned but could be more supported by empirical or theoretical rationale.

Response: We thank the reviewer for noting that our motivation for using the Wasserstein distance in domain adaptation was under-explained. In the revised manuscript, we expand the rationale in the **Multi-Granularity Contrastive Learning and Domain Adaptation** subsection by clarifying why the Wasserstein-1 distance can provide more stable and informative gradients for aligning source and target feature distributions, especially when the two distributions have limited overlap under large domain shifts.

The following are the revisions made in the **Multi-Granularity Contrastive Learning and Domain Adaptation** subsection to provide stronger theoretical and empirical motivation for using the Wasserstein distance in domain adaptation, and to clarify how it contributes to stable and effective cross-domain alignment in our overall optimization objective.

We choose the Wasserstein-1 distance for domain alignment because it provides a well-behaved discrepancy measure with informative gradients even when the source and target feature distributions have limited overlap under large domain shifts. In contrast to the Jensen–Shannon divergence commonly used in GAN-based alignment, which may lead to vanishing gradients when the two distributions are far apart, Wasserstein-1 directly reflects the optimal transport cost required to move probability mass from the source latent space to the target latent space, which typically yields more stable optimization. This property is particularly important in our setting, where the latent representations learned from heterogeneous IoT environments may exhibit substantial mismatch. Following the standard formulation of Wasserstein learning, we implement the objective with a gradient-penalized critic network to enforce the Lipschitz constraint, which further stabilizes training, improves reproducibility, and ultimately yields more reliable cross-domain alignment in the latent space [5, 6].

The use of two benchmark IoT traffic datasets with no reliance on labeled target anomaly data is appropriate and reflects real-world constraints. However, including additional baselines—especially recent state-of-the-art unsupervised domain adaptation methods—would strengthen empirical comparisons beyond autoencoder and variational autoencoder methods.

Response: We thank the reviewer for this valuable suggestion. In the revised manuscript, we will extend the experimental evaluation by adding an additional benchmark IoT traffic dataset, **ToN_IoT** [7], in the **Experiments** section. Specifically, we will incorporate ToN_IoT into our cross-domain evaluation protocol alongside WUSTL-IIOT-2021 and ACI-IoT-2023, and report the corresponding quantitative results to further demonstrate the robustness and generality of CDA-ADN across diverse IoT environments.

The paper mentions improvements in metrics such as Matthews correlation coefficient and sensitivity but should also report specificity and F1-score for a more holistic evaluation.

Response: We thank the reviewer for this valuable suggestion regarding the evaluation protocol. We agree that relying only on Accuracy, MCC and Sensitivity does not fully capture the behaviour of the detectors under class imbalance. In the revised manuscript, we have updated the main cross-domain results in the **Experimental Results and Analysis** subsection so that, for each source-to-target transfer setting, we additionally report **Specificity** and **F1-score** for all compared methods, together with AUC-ROC and AUC-PR. Representative results for the WUSTL-IIoT-2021 → ACI-IoT-2023 and WUSTL-IIoT-2021 → ToN_IoT transfer scenarios are shown in Tables 6 and 7.

Table 6: Cross-domain detection performance from WUSTL-IIoT-2021 (source) to ACI-IoT-2023 (target). Values are reported as mean ± standard deviation over five independent runs.

Model	Accuracy (%)	MCC	Sensitivity	Specificity	F1-score	AUC-ROC	AUC-PR
AE	76.5 ± 1.2	0.52 ± 0.03	0.57 ± 0.05	0.82 ± 0.02	0.48 ± 0.04	0.75 ± 0.03	0.28 ± 0.05
VAE	81.3 ± 1.5	0.62 ± 0.04	0.66 ± 0.06	0.86 ± 0.02	0.57 ± 0.04	0.87 ± 0.02	0.51 ± 0.04
AT	87.5 ± 1.0	0.70 ± 0.03	0.79 ± 0.04	0.89 ± 0.02	0.67 ± 0.03	0.90 ± 0.02	0.58 ± 0.04
GDN	88.6 ± 0.9	0.73 ± 0.03	0.81 ± 0.04	0.90 ± 0.02	0.69 ± 0.03	0.91 ± 0.01	0.60 ± 0.03
CDA-ADN	92.8 ± 0.7	0.80 ± 0.02	0.88 ± 0.03	0.93 ± 0.01	0.76 ± 0.03	0.92 ± 0.01	0.64 ± 0.03

Table 7: Cross-domain detection performance from WUSTL-IIoT-2021 (source) to ToN_IoT (target). Values are reported as mean ± standard deviation over five independent runs.

Model	Accuracy (%)	MCC	Sensitivity	Specificity	F1-score	AUC-ROC	AUC-PR
AE	69.8 ± 1.6	0.47 ± 0.03	0.55 ± 0.05	0.80 ± 0.03	0.44 ± 0.04	0.72 ± 0.03	0.26 ± 0.04
VAE	75.2 ± 1.7	0.58 ± 0.04	0.63 ± 0.05	0.85 ± 0.02	0.54 ± 0.04	0.84 ± 0.02	0.48 ± 0.04
AT	81.0 ± 1.3	0.66 ± 0.03	0.75 ± 0.04	0.88 ± 0.02	0.62 ± 0.03	0.88 ± 0.02	0.55 ± 0.03
GDN	82.1 ± 1.2	0.69 ± 0.03	0.77 ± 0.04	0.89 ± 0.02	0.64 ± 0.03	0.89 ± 0.02	0.57 ± 0.03
CDA-ADN	88.2 ± 0.9	0.77 ± 0.02	0.86 ± 0.03	0.92 ± 0.01	0.73 ± 0.03	0.91 ± 0.01	0.61 ± 0.03

It would add significant value to include ablation studies that individually assess contributions from: conditional variational encoding, dynamic adaptation layers, and multi-granularity contrastive learning components. This clarity will help justify design choices and pinpoint critical contributors to improved performance.

Response: We thank the reviewer for this helpful suggestion regarding the ablation study and fully agree that it is important to disentangle the contributions of all major components in CDA-ADN. In the revised manuscript, we have expanded the ablation experiment to individually assess the impact of the conditional variational encoder, the dynamic input–output adaptation layers, and the multi granularity contrastive learning module. The updated ablation table now reports results for the full CDA-ADN model, the variant without multi granularity contrastive learning, the variant without adaptation layers, the variant without the conditional variational encoder, and a baseline that removes both the contrastive module and the adaptation layers.

The following are the revisions made in the **Ablation Study** subsection to individually assess the contributions of the conditional variational encoder, the dynamic input–output adaptation layers, and the multi-granularity contrastive learning module, thereby clarifying the key drivers of the performance gains.

To evaluate the contributions of different components in CDA-ADN, we conduct an ablation study by systematically removing key modules and analyzing their impact on performance. Specifically, we assess the effects of removing the multi-granularity contrastive learning module, the dynamic input–output adaptation layers, and the conditional variational encoder, as well as a reduced configuration that removes both contrastive learning and adaptation layers. The results are presented in Table 8.

The results in Table 8 highlight that each component contributes meaningfully to cross-domain anomaly detection. Removing the multi-granularity contrastive learning module results in a clear drop in MCC from 0.80 to 0.73 and in sensitivity from 0.88 to 0.79. This suggests that contrastive learning enhances feature separability, making it easier to distinguish between normal and anomalous patterns under domain shift.

Removing adaptation layers also negatively impacts performance, as shown by the drop in MCC to 0.75 and sensitivity to 0.82. This confirms that the latent-guided input–output transformations effectively reduce cross-domain distribution mismatch and improve generalization to the target domain.

Table 8: Ablation study results on the target domain (mean ± std).

Configuration	MCC	Sensitivity
Full Model (CDA-ADN)	0.80 ± 0.02	0.88 ± 0.03
Without Multi-Granularity Contrastive Learning	0.73 ± 0.03	0.79 ± 0.04
Without Adaptation Layers	0.75 ± 0.02	0.82 ± 0.03
Without Conditional Variational Encoder	0.74 ± 0.03	0.80 ± 0.04
Without Contrastive Learning and Adaptation Layers	0.69 ± 0.04	0.75 ± 0.05

Replacing the conditional variational encoder with a deterministic GRU autoencoder leads to a noticeable drop in MCC and sensitivity as well, indicating that probabilistic latent modeling together with KL regularization helps learn a more structured and transferable latent space.

When both contrastive learning and adaptation layers are removed, the performance deterioration is more pronounced. The MCC score drops further to 0.69, and sensitivity decreases to 0.75, indicating a substantial degradation in the model's ability to detect anomalies. The absence of both components causes the model to rely mainly on reconstruction learning, which is insufficient for capturing domain-invariant yet discriminative features. This confirms that the contrastive module and adaptation layers provide complementary benefits and work synergistically to enhance model robustness under domain shift.

These findings underline the necessity of integrating conditional variational encoding, multi-granularity contrastive learning, and dynamic adaptation layers to achieve robust and generalizable anomaly detection. Removing any single component results in measurable degradation, and removing multiple components leads to a substantial decline in performance.

Additionally, sensitivity analysis on hyperparameters such as the number of target samples in the fine-tuning stage or latent dimension size could provide practical implementation guidance.

Response: We thank the reviewer for this practical suggestion. In addition to the sensitivity check on the contrastive-learning hyperparameters, the revised manuscript now includes a brief sensitivity analysis on two implementation-critical factors: the number K of unlabeled normal target-domain samples used in the fine-tuning stage and the latent dimensionality d_c of the conditional variational encoder. Specifically, in the **Sensitivity Analysis of Hyperparameters** subsection of the **Experiments** section, we discuss how increasing K from a very small value to a moderate number of normal target samples improves performance before saturating, and how choosing d_c in a mid-range yields a favorable trade-off between representation capacity and robustness.

The following are the revisions made in the **Sensitivity Analysis of Hyperparameters** subsection to provide practical guidance on the number of unlabeled normal target samples used in fine-tuning and the latent dimensionality of the conditional variational encoder.

In addition to contrastive-learning hyperparameters, we further examine two implementation-critical factors specific to CDA-ADN: the number K of unlabeled normal target-domain samples used in the fine-tuning stage and the latent dimensionality d_c of the conditional variational encoder. With $d_c = 64$ fixed, increasing K from 10 to 50 and 100 improves the target-domain accuracy from about 90.8% to 92.0% and 92.8%, while further increasing to $K = 200$ yields only a marginal gain to roughly 93.0%, suggesting that performance saturates once a modest amount of normal target data is available. Conversely, with $K = 100$ fixed, varying d_c in $\{16, 32, 64, 128\}$ results in accuracies of approximately 91.3%, 92.3%, 92.8%, and 92.4%, respectively. These trends indicate that CDA-ADN already performs strongly with K in the range of 50–100 and that d_c in the range of 32–64 provides a stable trade-off between representation capacity and robustness.

The framework assumes availability of "a small subset of normal samples from the target domain" for fine-tuning. It would be helpful to discuss scenarios where even these samples may be scarce or contaminated by anomalies and how CDA-ADN might behave under such conditions or be extended.

Response: We appreciate the reviewer's insightful comment about the realism of the target-domain sampling assumption. In our setting, CDA-ADN assumes access to a small subset of mostly normal target-domain samples that are used to calibrate the latent-guided adaptation layers. When this subset becomes extremely small, the adaptation step is underconstrained and the gains over source-only training diminish, so the model behaves closer to a source-only detector that still benefits from cross-domain contrastive regularization but with

reduced domain-specific refinement. If the target subset is mildly contaminated by anomalies, these samples are treated as normal during fine-tuning and may partially bias the adaptation layers, which can slightly reduce sensitivity to such anomalous patterns. In the revised manuscript, we explicitly discuss these scenarios and outline possible extensions, such as filtering out high–reconstruction-error target samples before adaptation or incorporating robust estimators in the adaptation loss, to mitigate the impact of contamination while preserving the unsupervised nature of the framework.

The following is the revision made in the **Training and Optimization Strategy** subsection to discuss the behavior of CDA-ADN under scarce or mildly contaminated target samples.

The above two-stage procedure assumes access to a small subset of mostly normal samples from the target domain that are used to calibrate the latent-guided adaptation layers. When this subset is extremely small, the adaptation parameters become underconstrained and the benefit over source-only training is reduced, so CDA-ADN behaves closer to a source-only detector that still leverages contrastive and Wasserstein regularization but with limited domain-specific refinement. In practice, the target subset may also contain a small fraction of anomalous samples. Since these samples are treated as normal during fine-tuning, they can bias the adapted feature statistics and slightly reduce sensitivity to those particular anomalous patterns. A natural extension is to combine the adaptation step with a simple robustness mechanism, for example by discarding target samples with very high reconstruction error before updating the adaptation layers or by using robust aggregation operators instead of plain averages when estimating target-domain statistics. Such strategies preserve the unsupervised nature of CDA-ADN while mitigating the impact of scarce or mildly contaminated target samples.

Some of the related work citations are old or generic. For example, references [2], [5], and [9] cover classical statistical and deep learning models. Including citations to very recent advances in unsupervised domain adaptation or anomaly detection (last 2 years) would keep the literature review current.

Response: We thank the reviewer for pointing out the need to better reflect recent advances in unsupervised domain adaptation and anomaly detection. In the revised manuscript, we have updated the **Related Work** section to complement the classical statistical and deep learning references with several recent contributions on deep time-series anomaly detection and cross-domain representation learning. In particular, we now cite recent surveys on deep learning–based time-series anomaly detection and graph neural networks for time-series analysis, as well as very recent contrastive and domain-adaptive models for time-series anomaly detection and domain adaptation. These additions place CDA-ADN more clearly in the context of the state of the art and highlight how our cross-domain, unsupervised framework differs from and complements these latest methods.

The following are the revisions made in the **Related Work** section to incorporate recent advances in unsupervised domain adaptation and anomaly detection and to complement the classical references.

In parallel, recent surveys have provided comprehensive overviews of deep learning methods for time-series anomaly detection and graph neural networks for time-series analysis, including anomaly detection tasks [8,9]. These works highlight the rapid development of representation learning techniques for time-series anomalies and motivate the need for models that remain robust under distribution shifts across domains.

More recent work on domain adaptation for time series under feature and label shifts, and on inter-domain sensor alignment for wearable multivariate time series, further emphasizes the importance of learning domain-invariant temporal representations in an unsupervised or label-efficient manner [10, 11].

Certain sections (e.g., methodology) could benefit from clearer figures or diagrams depicting the model architecture and training workflow to improve reader comprehension.

Response: We thank the reviewer for this helpful suggestion. In the revised manuscript, we add a clearer workflow diagram in the **Training and Optimization Strategy** subsection to visually summarize the two-stage training procedure and the adaptation process, thereby improving readability and reader comprehension.

The following are the revisions made in the **Training and Optimization Strategy** subsection to include the added training workflow figure.

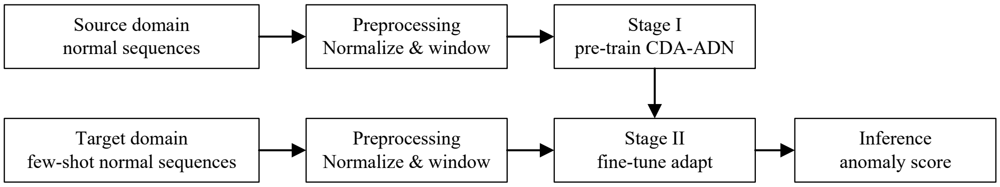

Fig. 1: Two-stage training workflow of CDA-ADN. Stage I pre-trains the model on source-domain normal traffic to learn general normal representations. Stage II performs lightweight adaptation on the target domain using only a small subset of normal samples, where only the domain-specific adaptation components are fine-tuned while the shared encoder-decoder is kept fixed. After adaptation, the model is directly applied to the target domain to compute anomaly scores and make anomaly decisions.

References

[1] M. Sakurada and T. Yairi, "Anomaly detection using autoencoders with nonlinear dimensionality reduction," in *Proceedings of the MLSDA 2014 2nd Workshop on Machine Learning for Sensory Data Analysis*, pp. 4–11, 2014.

[2] D. P. Kingma and M. Welling, "Auto-encoding variational bayes," *arXiv preprint arXiv:1312.6114*, 2013.

[3] J. Xu, H. Wu, J. Wang, and M. Long, "Anomaly transformer: Time series anomaly detection with association discrepancy," *arXiv preprint arXiv:2110.02642*, 2022.

[4] A. Deng and B. Hooi, "Graph neural network-based anomaly detection in multivariate time series," in *Proceedings of the AAAI conference on artificial intelligence*, vol. 35, pp. 4027–4035, 2021.

[5] M. Arjovsky, S. Chintala, and L. Bottou, "Wasserstein gan," *arXiv preprint arXiv:1701.07875*, 2017.

[6] I. Gulrajani, F. Ahmed, M. Arjovsky, V. Dumoulin, and A. C. Courville, "Improved training of wasserstein gans," *Advances in neural information processing systems*, vol. 30, 2017.

[7] N. Moustafa, "A new distributed architecture for evaluating ai-based security systems at the edge: Network ton_iot datasets," *Sustainable Cities and Society*, vol. 72, p. 102994, 2021.

[8] Z. Zamanzadeh Darban, G. I. Webb, S. Pan, C. Aggarwal, and M. Salehi, "Deep learning for time series anomaly detection: A survey," *ACM Computing Surveys*, vol. 57, no. 1, pp. 1–42, 2024.

[9] M. Jin, H. Y. Koh, Q. Wen, D. Zambon, C. Alippi, G. I. Webb, I. King, and S. Pan, "A survey on graph neural networks for time series: Forecasting, classification, imputation, and anomaly detection," *IEEE Transactions on Pattern Analysis and Machine Intelligence*, 2024.

[10] H. He, O. Queen, T. Koker, C. Cuevas, T. Tsiligkaridis, and M. Zitnik, "Domain adaptation for time series under feature and label shifts," in *International conference on machine learning*, pp. 12746–12774, PMLR, 2023.

[11] Y. Wang, Y. Xu, J. Yang, Z. Chen, M. Wu, X. Li, and L. Xie, "Sensor alignment for multivariate time-series unsupervised domain adaptation," in *Proceedings of the AAAI conference on artificial intelligence*, vol. 37, pp. 10253–10261, 2023.
